# The PIM family of oncoproteins: Small kinases with huge implications in myeloid leukemogenesis and as therapeutic targets

**DOI:** 10.18632/oncotarget.2330

**Published:** 2014-08-13

**Authors:** Kumar Saurabh, Michael T. Scherzer, Parag P. Shah, Alice S. Mims, William W. Lockwood, Andrew S. Kraft, Levi J. Beverly

**Affiliations:** ^1^ James Graham Brown Cancer Center, University of Louisville, Louisville, KY; ^2^ Department of Medicine, Division of Hematology and Oncology, University of Louisville School of Medicine, Louisville, KY; ^3^ Department of Pharmacology and Toxicology, University of Louisville School of Medicine, Louisville, KY; ^4^ Department of Bioengineering, J.B Speed School of Engineering, University of Louisville, Louisville, KY; ^5^ Hollings Cancer Center, Medical University of South Carolina, Charleston, SC; ^6^ Integrative Oncology, British Columbia Cancer Agency, Vancouver, British Columbia, Canada

**Keywords:** PIM-1, PIM-2, PIM-3, MYC, leukemia

## Abstract

PIM kinases are a family of serine/threonine kinases involved in cell survival and proliferation. There is significant structural similarity between the three PIM kinases (PIM1, PIM2 and PIM3) and few amino acid differences. Although, several studies have specifically monitored the role of PIM1 in tumorigenesis, much less is known about PIM2 and PIM3. Therefore, in this study we have used *in vitro* cell culture models and *in vivo* bone marrow infection/transplantation to assess the comparative signaling and oncogenic potential of each of the three PIM kinases. All three PIM kinases were able to protect FL5.12 cells from IL-3 withdrawal induced death. Interestingly, the downstream signaling cascades were indistinguishable between the three kinases. Transplantation of murine bone marrow co-expressing MYC and PIM1, PIM2 or PIM3 caused rapid and uniformly lethal myeloid leukemia. De-induction of MYC 18 days following transplantation significantly increased the survival of mice, even with continual expression of PIM kinases. Alternatively, mice treated at the pre-leukemic stage with a PIM kinase inhibitor increased the lifespan of the mice, even with continual expression of the MYC transgene. These data demonstrate the role of PIM kinases in driving myeloid leukemia, and as candidate molecules for therapy against human malignancies.

## INTRODUCTION

The PIM family of proteins are closely related serine/threonine kinases involved in cell survival, proliferation and apoptosis [1-3]. PIM1 was originally identified as a common site of Moloney murine leukemia proviral insertion [4-6]. Subsequently, numerous studies have been focused on identifying the role of PIM1 in driving leukemogenesis. The kinase family is composed of three members PIM1, PIM2 and PIM3, which are highly evolutionarily conserved in nearly all multicellular organisms. All three kinases are expressed in hematopoietic, neuronal, cardiomyocyte, endothelial, epithelial cell lineages and in embryonic stem cells [2, 4, 7-9]. In humans, PIM1 is located on chromosome 6, PIM2 on chromosome X and PIM3 on chromosome 22. Due to lack of obvious regulatory domains, regulation of their activity is at level of transcription, translation, and degradation [1, 2, 8, 10, 11]. Studies have also shown that at the translational level, PIM kinases are short lived due to the presence of AUUUA motifs at the 3'UTR. PIM kinases are weakly transcribed because of GC-rich motifs in their 5'UTR [6, 8].

Previous studies have shown that PIM1 cooperates with c-MYC and n-MYC during lymphomagenesis [4, 12]. Moreover, PIM1 collaborates with BCL2, GFI1 and E2A-PBX1 to promote lymphomagenesis [2, 4]. In cells lacking PIM1, activation of PIM2 through proviral insertion has been documented [13, 14]. Further, in cells lacking both PIM1 and PIM2 activation of PIM3 was observed, suggesting compensatory signaling through any of the three family members [15]. PIM kinases also mediate cell cycle regulators via phosphorylation such as cyclin-dependent kinase inhibitor 1 (CDK1A/B) and cell division cycle 25A (CDC25A/C) suggesting that PIM kinases partially share some substrates with other survival kinase pathways e.g. AKT [5, 9, 10, 16].

Overexpression of PIM kinases was discovered in human myeloid and T-cell leukemias and lymphomas, but recently over-expression has been described in numerous solid tumors (e.g. pancreatic and prostate cancers, non-small-cell lung cancer, squamous cell carcinoma, gastric carcinoma, liver carcinoma, liposarcoma etc.) [2, 4, 17]. Dysregulation of these proto-oncogenes in human cancer is typically not due to gene alterations, mutations or amplifications, but occurs as a result of regulation at the transcriptional level. The most common signaling activation is via NF-kB [9, 18, 19]. Studies have shown that inhibition of PIM1 using monoclonal antibodies or small molecule inhibitors can be effective at suppressing growth of tumor cells [20, 21]. Importantly, mice lacking PIM kinase family members are viable and fertile; indicating that potent inhibition of PIM kinases will provide a tolerable therapeutic window to inhibit tumorigenesis [15]. More than 100 PIM inhibitors have been described and some of these inhibitors are currently in clinical trials. Although most inhibitors are developed against PIM1, some have been shown to inhibit PIM2 and PIM3 [22-25]. Inhibitors such as AZD1208 are pan-kinase inhibitor currently in Phase I clinical trials [8].

Although several studies have monitored specifically the role of PIM1 in tumorigenesis, less is known about the role that PIM2 and PIM3 play in driving tumorigenesis. A comprehensive study comparing the three family members *in vivo* has not been previously performed. Therefore in this study we have examined the role of PIM1, PIM2 and PIM3 in driving myeloid leukemogenesis in cooperation with MYC using our mouse model [26]. We show that aggressive myeloid leukemia develops when any of the PIM kinases are expressed in mouse bone marrow in conjunction with MYC. Aggressive leukemia development requires the continued expression of MYC and the constitutive activity of PIM. Our data demonstrate the functional redundancy of each serine/threonine PIM kinase family member in driving myeloid leukemia and demonstrate the therapeutic benefit of targeting this family of kinases in leukemias that contain a high level of PIM kinase activity.

## RESULTS

### PIM family kinases are closely related and are upregulated in human acute myeloid leukemia (AML)

**Figure 1:**
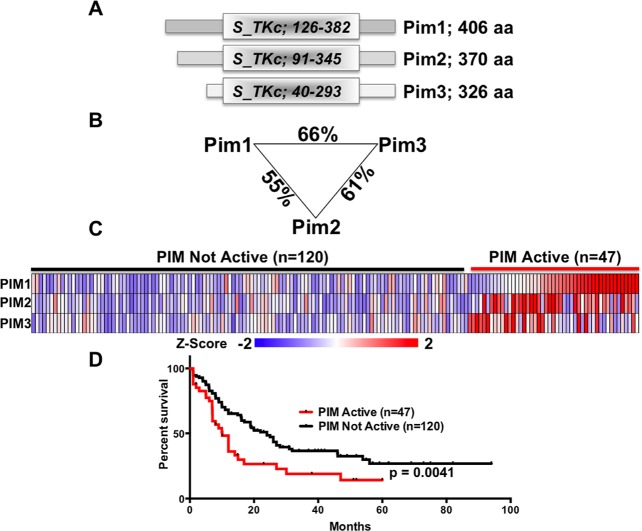
PIM kinases are a closely related family (A) Schematic comparing the domain structure of the PIM-family kinases. (B) Percentage of amino acid identity between the PIM-family kinases. (C) Expression data for 167 patients analyzed as part of the AML TCGA study were downloaded, Z-transformed and levels of PIM1, PIM2 and PIM3 determined. Tumors with a Z-Score >1 for any PIM gene were classified as ‘PIM active’ (n=47) whereas those without were classified as ‘PIM Not Active’ (n=120). (D) Survival data were linked to the samples in (C) and survival curves generated. Curves were compared using a Log-rank (Mantel-Cox) test and the resulting p-value of 0.0041 indicates a significant association between high PIM-family expression with poor survival.

To understand and compare the role of each PIM member plays in human acute myeloid leukemia, we describe the similarities between the three family members, including domain identification, peptide length, and amino acid sequence. PIM1 is 406 aa, PIM2 is 326 aa, and PIM3 is 370 aa (Figure 1a). Although the protein length is different, PIM isoforms contain highly similar kinase domain with high homology between the PIM family members (Figure 1b). To illustrate the importance of PIM family members in AML, we have performed analysis of AML patient samples to determine if expression of PIM family members occurs within the patients. 47 of the 167 leukemic patient samples analyzed from the TCGA dataset showed increased PIM family gene expression. A heat map of PIM expression and the corresponding groups is illustrated which demonstrates a clear distinction between the active and not active groups (Figure 1c). Further, survival distribution of patients with PIM kinase active was decreased compared to patients without similar PIM expression (Figure 1d), indicating PIM kinase's ability to contribute to poor survival in AML.

### PIM kinases protect cells in vitro

**Figure 2:**
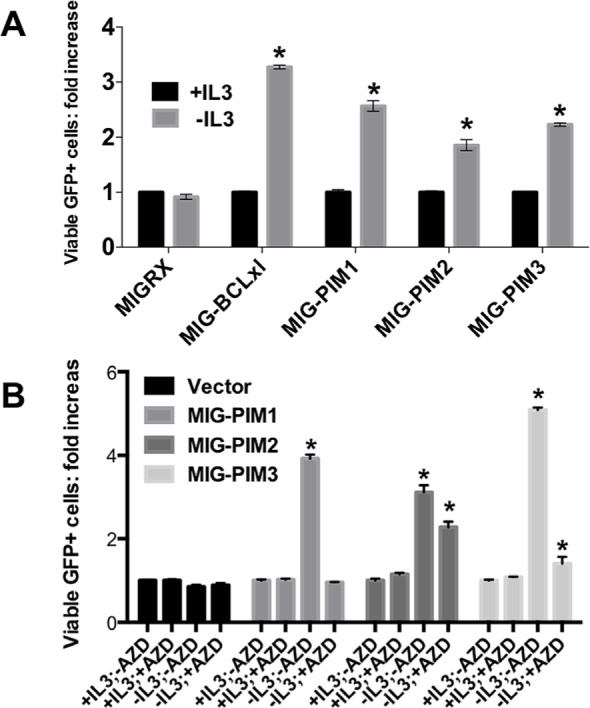
PIM-family kinases protect cells from death *in vitro* in a kinase dependent manner (A) PIM kinases protect FL5.12 cells from IL-3 withdrawal-induced death. Cells were infected with retroviruses expressing the indicated gene and GFP. 48 hours post-infection cells were either maintained in media containing IL-3 or were depleted of IL-3. 24 hours later, the relative number of GFP expressing cells was determined by FACS. (B) AZD1208 prevents PIM-induced cell survival in FL5.12 cells. The experiment was performed as shown in (A), however the PIM kinase inhibitor AZD1208 was added at the time of IL-3 removal.

Although each of the three PIM family kinases are closely related, a careful side-by-side comparison of their similarities and redundancies have not been performed. To assess whether each PIM family member can protect cells from cytokine withdrawal, we performed experiments using the IL-3 dependent FL5.12 murine pro-B-cell lymphoid cell line. FL5.12 cells were infected with retroviral constructs expressing individual PIM members and GFP. After 48 hours, IL-3 was withdrawn from the cells for 24 hours to determine if each PIM family members could maintain survival in the absence of IL-3. All three PIM kinase positive cell populations increased at least two-fold after IL-3 withdrawal, suggesting that each PIM isoform protects cells against IL-3 withdrawal in FL5.12 cells (Figure 2a). Further, we were interested in whether the pan-PIM Kinase inhibitor AZD1208 could halt the survival of PIM overexpressing cells after IL-3 withdrawal (Figure 2b). After 24 hours of IL-3 depletion and 1μM AZD1208 treatment, the survival advantage provided by each of the PIM family members was dramatically decreased, however to different extents. These data suggest that PIM kinase activity is required to increase survival in FL5.12 cells following IL-3 withdrawal.

### Comparison of *in vitro* biochemical properties of PIM1, PIM2 and PIM3

**Figure 3:**
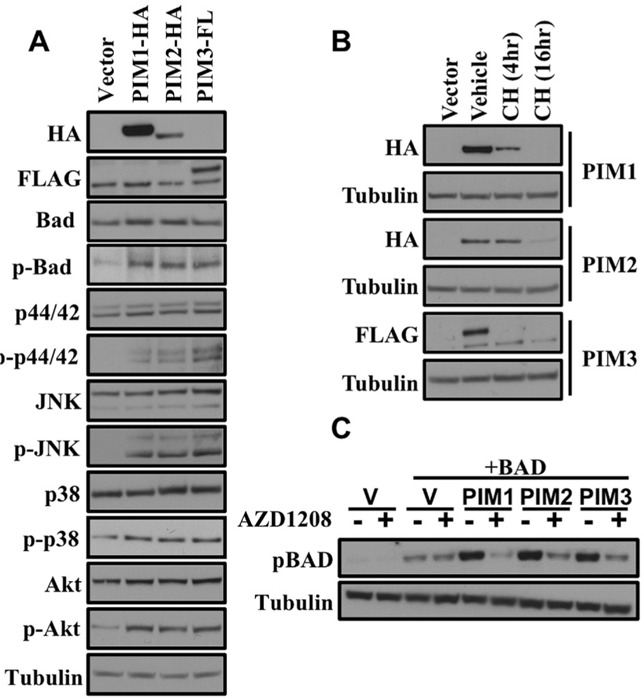
Comparison of *in vitro* biochemical properties of PIM1, PIM2 and PIM3 by western blot (A) Signaling pathway level comparison downstream of the PIM kinases in HEK293T cells. The indicated construct was transfected into HEK293T cells and 48 hours later cell lysates were prepared and western blots were performed with the indicated antibodies. (B) Protein stability comparison after cyclohexamide treatment. 48 hours post-transfection of HEK293T with the indicated constructs cells were treated with vehicle or cycloheximide for the indicated times. (C) Phosphorylation of BAD by PIM kinases is inhibited by AZD1208 treatment. 48 hours post-transfection of HEK293T cells with the indicated constructs cells were either treated with vehicle or 1 μM AZD1208. Lysates were prepared and western blots were performed with the indicated antibodies.

Expression of HA tagged PIM1 and PIM2 or FLAG tagged PIM3 led to increased phosphorylation of pro-apoptotic BAD and the MAPK family member JNK (Figure 3a). In this experiment, an increase in phosphorylated AKT and phosphorylated p44/42 was observed. PIM1 and PIM2 have similar half-lives of less than 16 hours while PIM3 was rapidly degraded by 4 hours (Figure 3b). AZD1208, a pan-PIM kinase inhibitor, was used to treat HEK293T cells that transiently express each PIM family member to determine the effect that the inhibitor has on the deactivation of pro-apoptotic BAD. After a 3 hour treatment of AZD1208 phosphorylated BAD was dramatically decreased (Figure 3c). These data indicate that AZD1208 inhibits the PIM-induced phosphorylation of BAD, thus enabling cells to succumb to apoptosis.

### Expression of MYC and PIM1, PIM2, or PIM3 causes rapid and lethal leukemia

**Figure 4:**
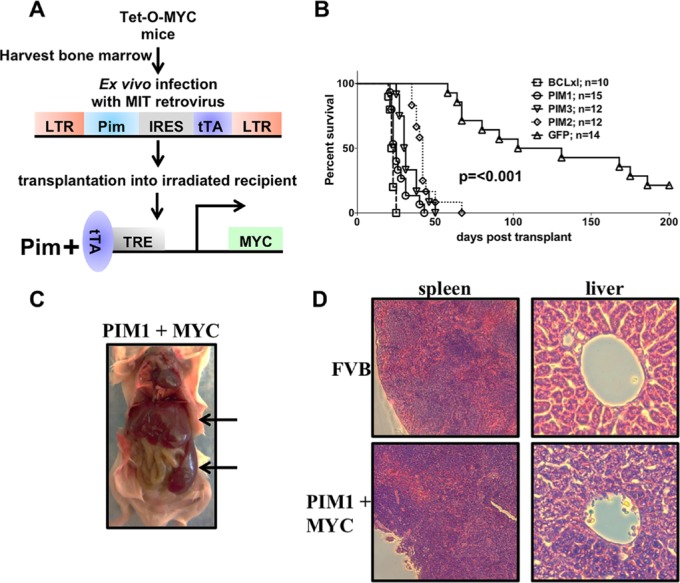
Co-expression of MYC and PIM1, PIM2 or PIM3 causes rapid and lethal leukemia (A) Schematic of *in vivo* bone marrow infection/reconstitution experiment. (B) Kaplan-Meyer curve of PIM+MYC *in vivo* experiment. Lethally irradiated FVB mice were injected with bone marrow cells expressing MYC and GFP, PIM1, PIM2, PIM3 or BCLxl. (C) Gross histology of PIM+MYC induced leukemia. Leukemia infiltrates spleen and liver; indicated with arrows (D) H&E stains of liver and spleen sections.

To understand the functional redundancy of the three PIM Family kinases, and to determine if the biologic similarities *in vitro* correspond to control of tumorigenesis, we performed *in vivo* assays. In mice all three PIM kinases, co-expressed with MYC, drastically accelerated myeloid leukemia. Retroviruses containing the sequence of each of the PIM kinases and a tetracycline trans-activator (tTA) were used to infect MYC transgenic bone marrow cells containing a tetracycline responsive element (TRE) upstream of MYC (Figure 4a). The infected bone marrow cells were then transplanted into irradiated FVB mice to express MYC and PIM1, PIM2 or PIM3 in the absence of tetracycline. Kaplan Meyer curve (Figure 4b), gross (Figure 4c), and histological analysis (Figure 4d) of the experimental mice indicate a burden of myeloid leukemia. All PIM family members, in combination with MYC, rapidly and lethally accelerated myeloid leukemia. All mice were euthanized when they exhibited hind limb paralysis, lethargy or an extremely distended abdomen. All PIM1 (n=15), PIM2 (n=12), and PIM3 (n=12) mice succumbed to disease by an average of 25, 42, and 30.5 days, respectively, whereas 50% of mice expressing only MYC succumb to disease at 130 days. These data indicate that the combination of PIM and MYC accelerate myeloid leukemia in mice. Mice receiving bone marrow expressing both BCLxl and MYC were used as a positive control. This combination was extremely potent and leads to complete penetrance of lethal myeloid leukemia by 25 days [27]. Gross analysis of a leukemic mouse post mortem also indicates a significant leukemic affliction by the development of enlarged spleen and liver (Figure 4c). Comparison between a normal FVB spleen and liver and a PIM1+MYC leukemic spleen and liver stained with H&E also demonstrated leukemic onset with an increase in cells stained with hematoxylin (purple) in the leukemic liver and spleen. To further prove that mice developed leukemia, we transplanted splenocytes into sub-lethally irradiated syngeneic recipients. All mice receiving primary leukemic splenocytes also succumbed to an aggressive myeloid leukemia (Supplemental figure 1). Thus, PIM family members accelerate leukemia in cooperation with MYC in this mouse model.

**Figure 5:**
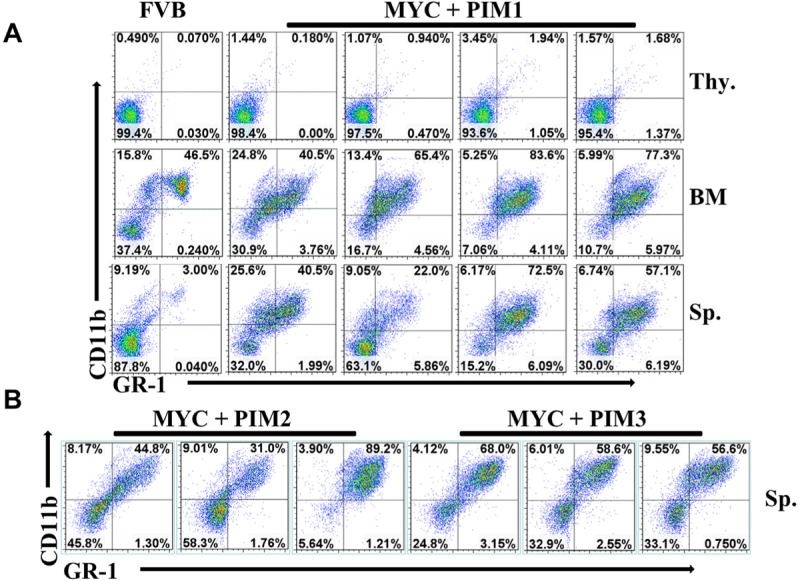
Co-expression of MYC and PIM kinases drives myeloid leukemia (A) Splenocytes (Sp.), thymocytes (Thy.) and bone marrow (BM) were prepared from four representative MYC + PIM1 mice and a non-manipulated FVB mouse. Cells were stained with the Ly-6G/Ly-6C (GR-1) and CD11b (Mac-1) antibodies and analyzed by FACS. (B) Comparison of splenocytes from 3 representative mice from each genotype; MYC + BCLxl, MYC + PIM1, MYC + PIM2, MYC + PIM3 stained with Ly-6G/Ly-6C (GR-1) and CD11b (Mac-1) antibodies and analyzed by FACS.

**Figure 6:**
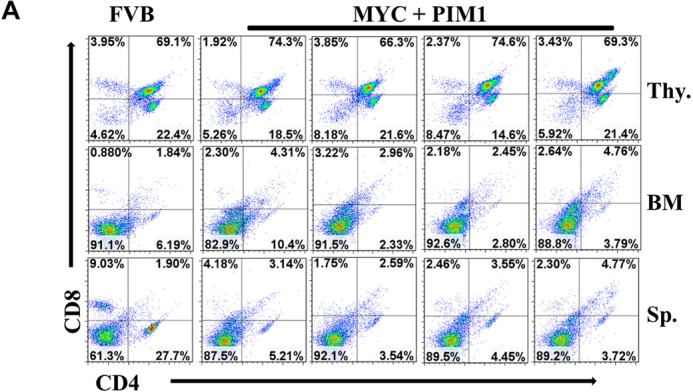
Co-expression of MYC and PIM1 does not affect T-cells Splenocytes (Sp.), thymocytes (Thy.) and bone marrow (BM) were prepared from four representative MYC + PIM1 mice and a non-manipulated FVB mouse. Cells were stained with CD4 and CD8 antibodies and analyzed by FACS.

To determine the type of leukemia that results from over expression in mice of PIM kinases and MYC co-expression, flow cytometry (FACS) analysis was employed with antibodies against myeloid and T-cell markers. FACS analysis of four PIM1/MYC mice and one mouse from PIM2, PIM3, and BCLxl cohorts demonstrates only myeloid cell (GR-1 and CD11b) markers, indicating a myeloid, not T-cell leukemia (Figure 5). The PIM family does not alter T-cells and was negative for T-cell markers CD4 and CD8, suggesting that the PIM kinases did not induce a T-cell leukemia in this model system (Figure 6). A significant population of GR-1/CD11b double positive cells was found in all PIM kinase experimental mouse spleens, indicating that these mice had myeloid leukemic burden (Supplemental figure 2).

### Inhibiting MYC at a pre-leukemic stage decreases the ability of PIM1, PIM2 or PIM3 to cause rapid leukemia onset

**Figure 7:**
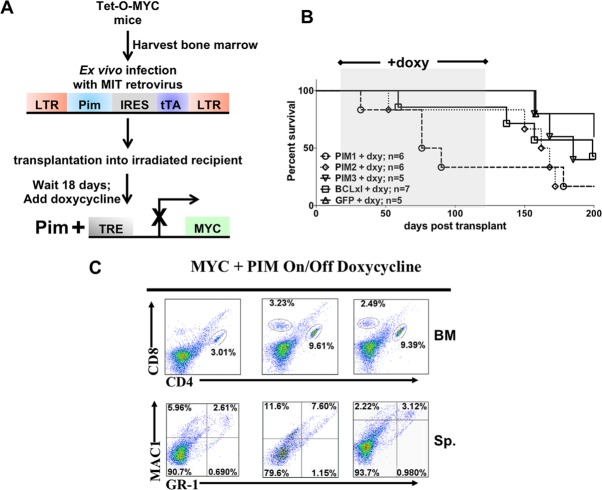
Inhibiting expression of MYC at a pre-leukemic stage decreases the ability of PIM1, PIM2 or PIM3 to cause rapid leukemia onset (A) Schematic of Tet-O-MYC bone marrow transplantation and doxycycline treatment experiment. 18 days following injection mice were switched to doxycycline containing chow to de-induce the MYC transgene. Following 100 days on doxycycline chow, the mice were switched back to regular feed. (B) Kaplan-Meyer curve of mice showing the window of doxycycline treatment (C) Representative FACS data showing population of T-cells in bone marrow.

To assess the requirement for continued MYC activity and whether PIM expression is sufficient to maintain leukemogenesis without the expression of MYC, we de-induced MYC expression at a pre-leukemic stage of cancer development in the mice by giving doxycycline containing feed. Six mice succumbed to disease during the window of MYC arrest. FACS analysis demonstrate that four of the six mice had a population of CD4 positive cells in the bone marrow (Figure 7). Furthermore, it is of interest to note that the majority of mice that succumbed on doxycycline were expressing PIM1. After 100 days on doxycycline chow (MYC off), the mice were switched back to normal feed. Most experimental subjects eventually succumbed to disease, although in many instances we were not able to detect aberrant populations of hematopoietic cells in these animals. All mice were euthanized and analyzed once they were on normal chow for an additional 100 days. These data are intriguing because it may suggest that in the absence of MYC expression PIM1 may be slightly more potent than the other two family members.

### Inhibition of PIM by AZD1208 halts MYC and PIM synergism *in vivo*

**Figure 8:**
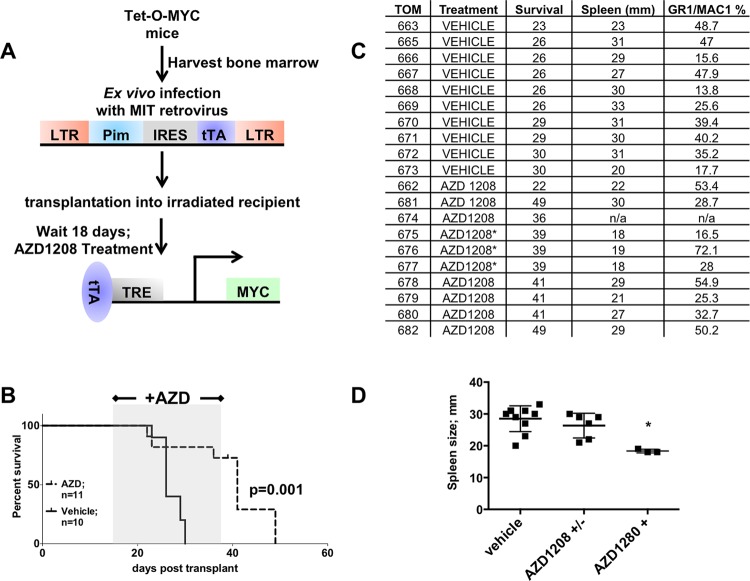
Treatment of mice expressing PIM1 and MYC with a PIM inhibitor extends life of mice (A) Schematic of Tet-O-MYC bone marrow transplantation and AZD1208 treatment experiment. 18 days following injection mice were given 30 mg/kg AZD1208 or vehicle daily by oral gavage for 20 days. (B) Kaplan Meyer curve showing AZD1208 treatment window. (C) PIM1 vehicle and AZD1208 treated spleen size and GR1/MAC1 positive cell population percentage in spleen. Mice indicated with an asterisk were sacrificed and analyzed 24 hours after the last AZD1208 treatment. These mice were not visible ill when sacrificed. (D) Graph depicting data from (C). Mice from vehicle treatment (vehicle) and AZD1208 group (AZD1208 +/−) were sacrificed when visibly ill, whereas the AZD1208+ group were euthanized 24 hours after last AZD1208 treatment and were not visible ill. Mice sacrificed from this last group had significantly smaller spleens compared to the AZD1208 mice never treated with drug, according to a one way ANOVA; p= < 0.005.

To determine if PIM activity is required to sustain leukemogenesis after a MYC/PIM pre-leukemic state, we treated mice daily with PIM inhibitor AZD1208. Efficacy of the pan-PIM kinase inhibitor AZD1208 was analyzed by treatment of 10 mice by oral gavage with the inhibitor and 10 mice treated daily with the vehicle. Vehicle treated mice succumbed to myeloid leukemia while inhibitor treated mice life span increased until treatment stopped after 20 days. 24 hours after the last AZD1208 treatment three random inhibitor treated mice were sacrificed to determine the extent of leukemia after treatment (Figure 8c & d* or AZD1208+, repectively). Although the spleen size was drastically different between vehicle and mice on AZD1208 (Figure 8b & c), the percentage of myeloid cells in the spleen still suggests a significant burden of leukemia in the inhibitor treated mice (Figure 8c). In fact, once AZD1208 treatment was stopped all mice eventually succumbed to disease and these mice had spleen sizes comparable to the vehicle treated mice previously analyzed from the same experiment (Figure 8d). Thus, treatment with a PIM kinase inhibitor extends the life of the mice, although it does not appear to cure the leukemia. Future work will determine if PIM kinase inhibition can be combined with additional therapeutic regimens to enhance cell death.

## DISCUSSION

In this study, for the first time we demonstrate the role of all three PIM kinases in driving tumorigenesis in both tissue culture and animal models. The expression of PIM kinases has been shown to be upregulated in several hematological malignancies and solid tumors [1, 2, 28]. Our MYC-driven mouse model shows that all three PIM members behave similarly in driving tumorigenesis by accelerating MYC-induced myeloid leukemia. Several previous studies were focused on the cooperation of MYC with PIM1 in mouse model in driving tumorigenesis [4, 12, 29] but less is known about the role of PIM2 and PIM3 in tumorigenesis. The levels of PIM1 has been shown to be elevated in human myeloid and T-cell leukemias and lymphomas but alteration in PIM kinase expression has also been identified in prostate, liver, bladder, pancreatic, gastric, head & neck squamous cell carcinoma, oral, and colorectal in human patients [2, 21, 30]. Our *in vivo* model reveals that all PIM kinase family members can cooperate with MYC to induce myeloid leukemia. Log-rank test of patients with AML also demonstrate that PIM oncogenes are highly expressed in approximately 30% of the samples analyzed, which correlate with poor survival in these patients. Closer analysis of the expression levels of individual PIM kinases suggests functional redundancy of the PIM family, such that there are very few samples that highly express more than one family member. This observation fits quite well with the data present herein that demonstrates functional redundancy of each family member *in vitro* and *in vivo*. Thus, all three PIM kinases are equally important as therapeutic targets.

We also demonstrate the individual oncogenic potency of PIM kinase family. Inhibiting MYC at a pre-leukemic state reveals the weaker oncogenic potency of kinases. These findings further support previous *in vitro* reports about the cooperation of PIM with other oncogene in the development of leukemia [2, 4, 24]. Several mice succumbed to disease during doxycycline treatment but did not have leukemic symptoms such as hind limb paralysis or enlarged spleen or livers. No significant myeloid cell populations were present, indicating that these mice did not develop typical myeloid leukemias, as seen previously. Possible explanations for this could be that doxycycline chow may not be able to fully inhibit MYC expression in several tissues, which would leave a small population of cells still expressing the potent combination of MYC and PIM. It is also interesting that several of these mice had significant populations of CD4 and CD8 positive cells in the bone marrow, suggesting a T-cell disease. However, conclusions remain unclear and require further investigation.

Previous reports have shown that PIM1 overexpression increased ERK1/2 phosphorylation in prostate cancer cells [29], increased p38-MAPK activation by phosphorylation in Basophils [31], and increased activation of JNK in cardiomyocytes by PIM3 [32]. Here, we have compared, for the first time, all PIM family members in regards to the expression of these known MAPK pathway members. Previous reports have also used the IL-3 dependent FL5.12 cells as a model for hematopoietic progenitor cells [33]. We used FL5.12 cells as a model to compare how each PIM member affected FL5.12 cell survival after IL-3 withdrawal. Data presented also demonstrate that all three PIM kinases can protect FL5.12 cells from IL-3 withdrawal. Further, when cells were treated with AZD1208 and IL-3 was withdrawn there was a dramatic decrease in the ability of the proteins to protect the survival of the cells. Interestingly, whereas AZD1208 completely abrogated the ability of PIM1 to protect the cells from IL-3 withdrawal, AZD1208 was less capable of blocking PIM2 and PIM3. In fact, these findings are consistent with the ability of AZD1208 to differentially inhibit the kinase activity of each of the family members, such that it is known that AZD1208 is most potent against PIM1, slightly less potent against PIM3 and even less potent against PIM2. Comparison of protein stability by cycloheximide in PIM kinases over-expressed HEK293T cells also proved valuable information regarding the protein stability of each PIM member and how this stability plays a role in leukemogenesis. These *in vitro* biochemical results show that all PIM family members activate the same signaling pathways (MAPK/JAK/STAT) and increase survival in FL5.12 cells.

Inhibition of PIM kinase with AZD1208, a Pan PIM kinase inhibitor, results in reduction of spleen size in mice which was in accordance to previous report showing decrease in cell proliferation in AML and xenograft mouse models [34]. It is possible that the reduction in spleen size may be because of its well documented suppression in phosphorylation of BAD which resulted in a reduction of PIM kinase mediated cell survival and shift towards apoptosis. These data also support the previous *in vitro* finding that AZD1208 causes reduction in cell size and cell number [15, 34] thus activating signaling event mediated apoptosis. In contrast, withdrawal of AZD1208 after 20 days of treatment results in relapse suggesting the myeloid cells that have survived are sufficient to recapitulate the leukemia in the presence of MYC. This result suggests that AZD1208 efficacy could be improved through combination therapy with standard chemotherapeutics.

In conclusion, our mouse model shows the regulation of all three PIM family members in the development of leukemogenesis with the average oncogenic latency being dependent upon which PIM family member is driving the disease. PIM kinases co-expressed with MYC have produced an aggressive myeloid leukemia in mice and silencing this family will increase chances of leukemic cells to initiate apoptosis. These finding underline the significance of PIM kinase family as a regulator of leukemia and the potential utility of a drug targeting this enzyme.

## MATERIALS & METHODS

### Plasmids

All epitope tagged PIM proteins were produced in the Kraft laboratory, Hollings Cancer Center, Medical University of South Carolina, Charleston and sub-cloned into the murine stem cell virus based retroviral vectors MIGRX and MITRX as described in our earlier study [35]. Clones were then transformed into 5-α competent *E. coli* cells (#C2987H, NEB, Ipswich, MA, USA) and DNA was prepped using eZNA kit as per the manufacturer's protocol (#D6924, Omega, Norcross, GA, USA).

### Cell culture and transfection

Human embryonic kidney 293T (HEK293T) cells and Phoenix-GP cells were procured from American Type Culture Collection (ATCC, Rockville, MD, USA) and cultured in DMEM medium (#SH30243.01, Hyclone, Logan, UT, USA) supplemented with 10% fetal bovine serum (#SH30070.03, Hyclone, Logan, UT, USA) and 1% antibiotic/antimycotic (#SV30010, Hyclone, Logan, UT, USA). FL5.12, a murine hematopoietic cell line, was a gift from Dr. Rathmell's lab, Department of Pharmacology and Cancer Biology, Sarah W. Stedman Nutrition and Metabolism Center, Duke University and cultured in RPMI (#SH30027.01, Hyclone, Logan, UT, USA) supplemented with 10% FBS, 1% antibiotic/antimycotic, 20 pM mouse recombinant IL-3 (#213-13, PeproTech, Rocky Hill, NJ, USA) and 55μM 2-mecaptoethanol (#21985-023, Gibco, Grand island, NY, USA). For IL-3 withdrawal experiments, FL.512 cells were washed with media without IL-3 and then suspended in fresh media with or without IL-3. DNA transfections were done with PIM constructs using polyethylenimine (#23966-2, Polysciences, Warrington, PA, USA) in Phoenix-GP cells and viruses were collected as described [26].

### Protein estimation and Western Blot

After transfection/infection cells were lysed with 1% CHAPS lysis buffer and protein were estimated as described in our previous study [36]. Western blots were performed in Bolt Bis-Tris gels (#BG4120BOX, Life Technologies, Grand island, NY, USA) as per manufacturers protocol using antibodies from Sigma-Aldrich, Cleveland, OH, USA (Tubulin #B512, FLAG poly-clonal #F7425); Roche, Nutley, NJ, USA (HA #3F10) and Cell Signaling, USA (p-Bad #9296 Bad #9292 p-p44/42 MAPK #4370, p44/42 MAPK #4695, p-p38 MAPK #4511, p38 MAPK #8690), p-SAPK/JNK #4668, SAPK/JNK #9258).

### Bone marrow harvest, infection and transplantation

*In vivo* experiments were done as described previously [26, 27, 35]. Briefly, bone marrow was flushed from untreated donor mice that carried the Tet-O-MYC transgene. Red blood cells were lysed and viral infections were then carried out in the presence of polybrene (#TR-1003-G, Millipore, Danvers, MA, USA) using retroviral PIM construct. After infection, cells were immediately transplanted into the tail vein of lethally irradiated FVB/n recipients acquired from Taconic (Figure 1d). For doxycycline treatment, normal mouse chow was swapped with doxycycline containing chow (#TD.00426, Harlan Laboratories, Madison, WI, USA) after 18 days of bone marrow transplantation (Figure 7a). For hematoxylin and eosin (H&E) staining tissue samples were shipped to Histoserve, Germantown, MD, USA. All experiments have been approved with the Institutional Animal Care and Use Committee (IACUC # 11090) of University of Louisville.

### Flowcytometry

Spleen, bone marrow and thymus were analyzed by FACS. In brief, single cells were isolated from tissues; red blood cells were lysed and blocked for 10 mins at room temperature with Fc Block (#553142 BD Biosciences, Miami, FL, USA). The samples were then stained with antibodies from BD Biosciences, Miami, FL, USA (Ly-6G & Ly-6C #553128; CD11b #552850; TER-119 #557909; CD3e #557984; CD4 #553730; CD8a #553036; CD45R #557957) eBioscience Inc., USA (CD150 #17-1501-81) for 30 mins at 4^o^C and then analyzed on Becton Dickinson FACScan with FlowJo.

### PIM expression in AML and survival analyses

PIM1, PIM2 and PIM3 RNA-Seq (RPKM) data for AML tumors from the Provisional TCGA dataset were downloaded from cBioPortal along with the corresponding clinical data on July 16, 2014. Expression for each gene was z-transformed across the dataset and samples with complete survival data used for subsequent analyses. Samples with a z-score >1 indicating the highest expressing samples - for one or more PIM gene were then identified and classified as ‘PIM Active’ where the remaining cases were classified as ‘PIM Not Active’. Heatmaps were generated using z-score data and plotted using Genesis software [37]. Survival curves were generated in Prism Graphpad and the Log-rank (Mantel-Cox) use to test for significant with a p<0.01 deemed significant.

### AZD1208 Treatments of mice

18 days after injection of cells expressing PIM1 and tTA, mice were randomly divided into two cohorts. One cohort of mice was treated daily for 20 days with 30 mg/kg of body weight with AZD1208 by oral gavage and the second cohort was treated daily for 20 days with an equal volume of vehicle (0.5% Hydroxypropyl methyl cellulose/0.1% Tween-80). Mice were treated every day for 20 days (Figure 8a).

## SUPPLEMENTARY MATERIAL AND FIGURES


