# Supplementary material for: The PIM family of oncoproteins: Small kinases with huge implications in myeloid leukemogenesis and as therapeutic targets
Source: Oncotarget. 2014 Aug 13;5(18):8503–14. doi: 10.18632/oncotarget.2330 (PMC4226700; doi:10.18632/oncotarget.2330)
Supplement: Supplementary file 1 [file oncotarget-05-8503-s001.pdf]

The PIM family of oncoproteins: small kinases with huge implications in myeloid leukemogenesis and as therapeutic targets

Supplementary Material

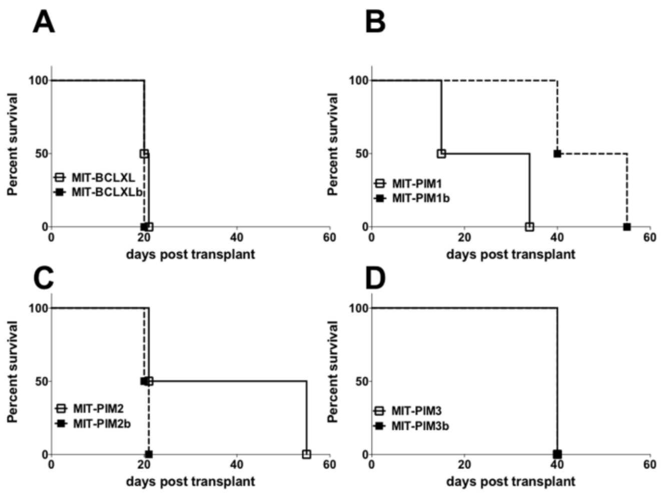

Supplemental figure 1: Myeloid malignancies expressing MYC and PIM1, PIM2 or PIM3 are transplantable leukemias. (A) BCL-XL (B) PIM1 (C) PIM2 (D) PIM3

Single cells suspensions from spleens of primary leukemic mice were frozen in complete media containing 10% DMSO at the time of analysis. Two vials representing two independent primary leukemias were thawed from each genotype and 2 X 10^6 viable cells were transplanted into the tail veins of sub-lethally irradiated FVB mice. Each leukemia was transplanted into two individual mice. Mice were monitored for disease following transplantation

	Survival (Days)	Splenocytes (% Total)			
**BCLxl +MYC**		GR1/ Mac1	CD4	CD8	CD4/CD8
**TOM530**	*20*	42			
**TOM532**	*22*	35			
**TOM533**	*22*	65			
**TOM536**	*23*	57	11		
**TOM566**	*21*	39			
**TOM568**	*22*	32			
**TOM569**	*25*	60			
**TOM570**	*25*	68			
**GFP + MYC**					
**TOM556**	*64*	20	28		
**TOM558**	*67*	10	16		
**TOM559**	*67*	25			
**TOM584**	*91*				
**TOM509**	*103*				
**TOM555**	*175*				
**PIM2 + MYC**					
**TOM543**	*35*	76			
**TOM544**	*35*	26			16
**TOM545**	*40*	31			
**TOM550**	*44*	47	15		
**TOM581**	*38*	41	15		
**TOM582**	*38*	26	32		
**TOM585**	*42*	16	20		
**TOM586**	*42*	50	11		
**TOM587**	*42*	16	37		
**TOM588**	*42*	32			

	Survival (Days)	Splenocytes (% Total)			
**PIM3 + MYC**		GR1/ Mac1	CD4	CD8	CD4/CD8
**TOM540**	*27*	47			
**TOM541**	*27*	53			
**TOM547**	*30*	54			
**TOM548**	*30*	33			
**TOM549**	*30*	64			
**TOM573**	*25*	45			
**TOM574**	*31*	46			
**TOM575**	*31*	30	11		26
**TOM579**	*38*	45			
**TOM580**	*38*	20			
**PIM1 + MYC**					
**TOM428**	*23*	31			
**TOM431**	*26*	65			
**TOM437**	*31*	80			
**TOM438**	*31*	64			
**TOM534**	*22*	42			
**TOM535**	*22*	45			
**TOM537**	*23*	28			
**TOM538**	*23*	25			
**TOM539**	*23*	33			
**TOM567**	*21*	24			
**TOM571**	*25*	30			
**TOM572**	*25*	48			

Supplemental Figure 2: Table showing percentages of hematopoietic lineages from the spleens of leukemic mice.
